# A note on the graphical presentation of prediction intervals in random-effects meta-analyses

**DOI:** 10.1186/2046-4053-1-34

**Published:** 2012-07-28

**Authors:** Charlotte Guddat, Ulrich Grouven, Ralf Bender, Guido Skipka

**Affiliations:** 1 Department of Medical Biometry, Institute for Quality and Efficiency in Health Care (IQWiG), Im Mediapark 8, Cologne, 50670, Germany; 2 Hannover Medical School, Hannover, Germany; 3 Faculty of Medicine, University of Cologne, Cologne, Germany

**Keywords:** Meta-analysis, Heterogeneity, Random effects model, Forest plot, Prediction interval

## Abstract

**Background:**

Meta-analysis is used to combine the results of several related studies. Two different models are generally applied: the fixed-effect (FE) and random-effects (RE) models. Although the two approaches estimate different parameters (that is, the true effect versus the expected value of the distribution of true effects) in practice, the graphical presentation of results is the same for both models. This means that in forest plots of RE meta-analyses, no estimate of the between-study variation is usually given graphically, even though it provides important information about the heterogeneity between the study effect sizes.

**Findings:**

In addition to the point estimate of the between-study variation, a prediction interval (PI) can be used to determine the degree of heterogeneity, as it provides a region in which about 95% of the true study effects are expected to be found. To distinguish between the confidence interval (CI) for the average effect and the PI, it may also be helpful to include the latter interval in forest plots. We propose a new graphical presentation of the PI; in our method, the summary statistics in forest plots of RE meta-analyses include an additional row, ‘95% prediction interval’, and the PI itself is presented in the form of a rectangle below the usual diamond illustrating the estimated average effect and its CI. We then compare this new graphical presentation of PIs with previous proposals by other authors. The way the PI is presented in forest plots is crucial. In previous proposals, the distinction between the CI and the PI has not been made clear, as both intervals have been illustrated either by a diamond or by extra lines added to the diamond, which may result in misinterpretation.

**Conclusions:**

To distinguish graphically between the results of an FE and those of an RE meta-analysis, it is helpful to extend forest plots of the latter approach by including the PI. Clear presentation of the PI is necessary to avoid confusion with the CI of the average effect estimate.

## Background

Using meta-analyses, the results of *k* studies related to the same question can be combined to produce an average result. For example, in the context of clinical trials comparing a new pharmaceutical with a placebo, the treatment effect in each trial may be quantified by the odds ratio. Each of the *k* effect estimates is recorded and finally summarized to one average estimate.

There are two different approaches in meta-analysis. The fixed-effects (FE) model assumes that the same treatment effect, *θ*, underlies all studies. Different estimates ${\overset{\wedge }{\theta }}_{1},\ldots ,{\overset{\wedge }{\theta }}_{k}$ for the true effect *θ*, resulting from the *k* studies are expected to arise solely from sampling error. By contrast, the random-effects (RE) model incorporates the between-study variation, taking into account the heterogeneous true effects $\theta_{1},\ldots ,\theta_{k}$[1]. This model is appropriate when the observed treatment effects between studies differ more from each other than would be expected from within-study variation alone. This heterogeneity between studies may arise from diversity in participants or interventions. The FE model can be viewed as a special case of the RE model, in which the between-study variation is 0.

The parameter to be estimated depends on the approach chosen. Under the assumption of a FE one true effect is estimated, whereas under the assumption of RE, the expected value *θ* of the distribution of true effects is estimated. Despite this difference between the two approaches, both the graphical presentation and the interpretation of the results are in practice the same for both models. The point and interval estimates of *θ* are commonly displayed in a forest plot as a diamond, irrespective of the model chosen [2-4]. Commonly used software packages in systematic reviews (for example, RevMan [5] in Cochrane reviews) do not distinguish between the two models in the graphical presentation of results. Apart from a numerical value, the estimate of the between-study variation, *τ*, is not shown in forest plots of RE models.

The use of the prediction interval (PI) has recently been proposed to illustrate the degree of heterogeneity in forests plots of RE meta-analyses [6-8]. A PI provides a predicted range for the true treatment effect in an individual study. Higgins *et al*. [7] proposed using an additional hollow diamond for the presentation of PIs, whereas Riley *et al*. [8] added extra lines to the usual diamond of the effect estimate and its CI. For explanatory purposes, Borenstein *et al*. [9] displayed a bell-shaped curve, truncated at the limits of the PI, in accordance with the assumption of normally distributed effects; however, to display the PI, they adopted the same graphical approach as the one proposed by Riley *et al*. [8].

In this paper, we propose a new graphical approach for the presentation of PIs based on the original suggestion by Skipka [6], and compare it with the approaches of Higgins *et al*. [7] and Riley *et al*. [8].

## Methods

### Addressing heterogeneity in random-effects meta-analysis

The RE model assumes differences in the treatment effects $\theta_{i}$ across *k* studies. Hence, the estimation and presentation of the average effect and its CI alone are insufficient. It is also important to quantify the heterogeneity between the effect sizes. The following measures are often used for this purpose: the between-study variance $\tau^{2}$, which can be estimated by various methods [10,11]; the *Q* statistic, which is a measure of weighted squared deviations; or *I*^*2*^, which describes the proportion of the total variance of the study effects due to heterogeneity [1,12,13]. One way to present the dispersion of the study effects graphically is to add the PI to the forest plot of RE meta-analyses.

Under the assumption that both the RE and the estimated average effect are approximately normally distributed, that is:

(1) $$\theta_{i}∼N\left(\theta ,\tau^{2}\right),\overset{\wedge }{\theta }∼N\left(\theta ,SE{\left(\overset{\wedge }{\theta }\right)}^{2}\right)\text{,}$$

Higgins *et al*. [7] suggest that the PI is:

(2) $$\left[\overset{\wedge }{\theta }-t_{1-\alpha /2;k-2}\sqrt{{\overset{\wedge }{\tau }}^{2}+\overset{\wedge }{SE}}{\left(\overset{\wedge }{\theta }\right)}^{2};\theta +t_{1-\alpha /2;k-2}\sqrt{{\overset{\wedge }{\tau }}^{2}+\overset{\wedge }{SE}}{\left(\overset{\wedge }{\theta }\right)}^{2}\right],$$

where $t_{1-\alpha /2;k-2}$ is the (1 − α/2) quantile of the *t*-distribution with *k-2* degrees of freedom, and $\overset{\wedge }{\tau }$ and $\overset{\wedge }{SE}\left(\overset{\wedge }{\theta }\right)$ denote the estimated between-study variation and the standard error of $\overset{\wedge }{\theta }$ respectively. Applying a *t*-distribution instead of a normal distribution reflects the uncertainty resulting from the estimation of *τ*.

However, the assumption that the true effects are normally distributed may not be justified. In these situations the choice of a different distribution [14] may be appropriate, leading to a different PI.

In contrast to the commonly presented CI, which quantifies the precision of the estimated average effect, the PI includes the effect of an individual study, with the level of confidence (1 − α). It is important to note that the PI provides no information on the statistical significance of $\overset{\wedge }{\theta }$.

The PI should be presented graphically in the forest plot of the RE meta-analyses. In such an extended forest plot, the degree of heterogeneity is illustrated, and a clear visual distinction is made between the results of the FE and the RE meta-analyses.

### Modified extension of the forest plot

Forest plots are a graphical presentation of the results derived from a meta-analysis. They allow a rapid overview of the potential heterogeneity of the studies analyzed. In conventional forest plots, the effect measures of the *k* studies with the corresponding CI are represented by a square with horizontal lines, in which the size of the squares reflects the weight that each study contributes to the meta-analysis. Below the results of the individual studies, the average estimate and its CI are displayed as a diamond, whose centre (vertical line) indicates the point estimate and whose width indicates the CI.

**Figure 1:**
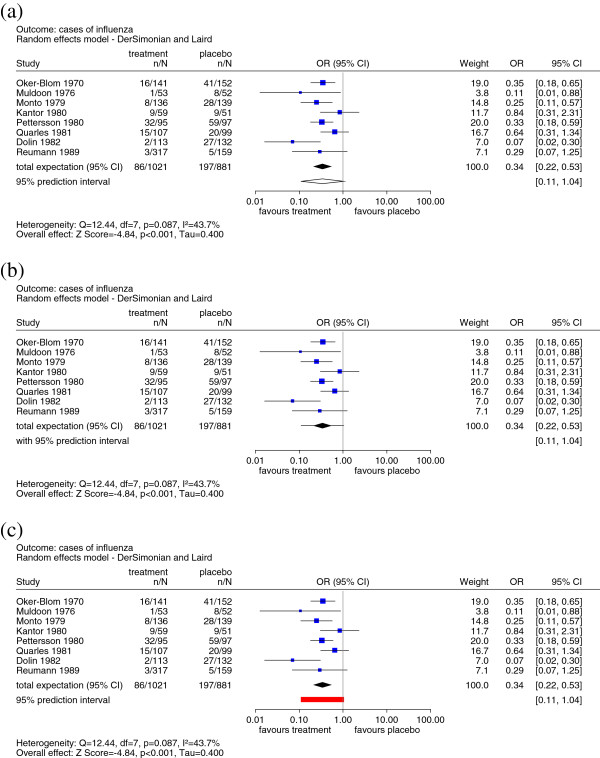
**a) Implementation of the prediction interval in forest plots as suggested by Higgins *et al* .**[7]**.b**) Implementation of the prediction interval in forest plots as suggested by Riley *et al*. [8]. **c**) New implementation of the prediction interval in forest plots. Meta-analysis of eight trials of amantadine for prevention of influenza [15].

To date, the PI has not been part of the common layout of forest plots: However, some proposals to include PIs have been made. Figure 1a shows the proposal by Higgins *et al*. [7], in which the PI is illustrated as a hollow diamond. Riley *et al*. [8] suggest a different presentation in which the confidence and PIs are ‘merged’ (Figure 1b). The point estimate and CI are shown in the typical form of a diamond, and are then extended by lines on both sides representing the width of the PI. Borenstein *et al*. [9] displayed the PI in the same way and, for explanatory purposes, added a truncated bell-shaped curve based on the assumption of a normal distribution.

We propose an alternative graphical approach based on the original suggestion by Skipka [6], which can be considered a mixture of the approaches described. As described previously, the row ‘total expectation (95% CI)’ represents the point and interval estimates for *θ* in the form of a diamond. We have added a new row, ‘95% prediction interval’, to the forest plot, illustrating the corresponding interval in an easily distinguishable way in the form of a rectangle (Figure 1c).

## Results and discussion

The importance of the PI as a method to incorporate heterogeneity in the presentation of RE meta-analyses has been discussed recently [16]. However, no transparent standard exists as to how to include PIs in forest plots. Higgins *et al*. [7] displayed the PI in the form of a diamond; that is, using the same symbol used to present the CI for the average effect. However, it could be misleading to use the same symbol for two different intervals. Riley *et al*. [8] suggested plotting a combination of both intervals by adding extra lines to the left and right end of the diamond representing the average effect and its CI. This method of illustration makes it even more difficult to distinguish between the CI and PI, and in addition, a line is already commonly used to present a CI.

To avoid such confusion, we recommend presenting the two intervals separately using different symbols. We suggest presenting the PI in an additional row of the forest plot in the form of a rectangle as originally proposed by Skipka [6]. The rationale for this is as follows: If we think of a set of infinitely large studies symbolized by squares in the corresponding forest plot, as already described, then because of the size of the studies, the horizontal lines representing the CIs converge towards zero. The dispersion of the study effects becomes visible when the squares are merged, leading to the form of a rectangle. In contrast to the illustration by Borenstein *et al*. [9], this representation of the PI can be used for any distributional assumption whereas the bell-shaped curve can be applied only under the assumption of a normal distribution.

Note that Skipka [6] used the term ‘heterogeneity interval’, rather than ‘PI’, because the interval describes the degree of heterogeneity between the studies. However, because the interval provides the region in which, with high confidence, the true effect measure of a new study lies, the term ‘prediction interval’ may be more appropriate. Hunter and Schmidt [17] proposed a further term for what can be regarded as the predecessor of the PI; in the context of psychometric meta-analysis, they adopted the term ‘credibility interval’, which ‘contains the distribution of true effects and is roughly analogous to the prediction interval’ [9].

We show the result of the same RE meta-analysis of eight studies investigating the effect of amantadine for the prevention of influenza [15], using the three approaches to present PIs described above (Figure 1). The estimated overall effect measured by the odds ratio for cases of influenza is 0.34, with a 95% CI ranging from 0.22 to 0.53. This interval is a measure of the precision of the expected value of the distribution of true effects, and it depends heavily on the number of studies included in the meta-analysis. It would be incorrect to conclude that the effect in a newly conducted study will be within the interval of 0.22 and 0.53 with 95% confidence. On the other hand, the PI provides information about the distribution of effects. In this example, the results are heterogeneous, with $\overset{\wedge }{\tau }=0.4$ (DerSimonian and Laird estimator [18]). The effect of a new study will be within an interval of 0.11 and 1.04 with 95% confidence. This is clearly shown in Figure 1c, thus avoiding misinterpretation, and represents our recommendation for the implementation of the PI in forest plots.

## Conclusions

The investigation of potential heterogeneity is an important task in meta-analysis. Various measures and statistical tests to assess heterogeneity have been suggested in the past [1,12,13]. Unfortunately, conventional forest plots fail to graphically present any measures related to heterogeneity. In addition, presenting the results of FE and RE models in the same way may convey the (incorrect) impression that the two models estimate the same parameter. The inclusion of the PI in the graphical presentation of RE meta-analyses provides additional information about the variation of treatment effects. To graphically distinguish between the CI and PI in forest plots, it is important to choose a different form of illustration, for example, a rectangle instead of a diamond, as the latter symbol is commonly used to present the average effect and its CI.

## Competing interests

The authors declare that they have no competing interests.

## Authors’ contributions

GS wrote the initial draft of the manuscript, and CG, UG, and RB wrote the final manuscript. All authors read and approved the final version.
